# Supplementary material for: Rapid turnover of life-cycle-related genes in the brown algae
Source: Genome Biol. 2019 Feb 14;20:35. doi: 10.1186/s13059-019-1630-6 (PMC6374913; doi:10.1186/s13059-019-1630-6)
Supplement: Supplementary file 3 — Protein alignments, corresponding Gblocks html file and CDS sequences for the lineage conserved gametophyte-biased genes. (ZIP 2109 kb) [file 13059_2019_1630_MOESM3_ESM.zip › Con_GA_alignments/SJ15142.aa_ali.fasta-gb.htm]

SJ15142.aa.fasta.trans.aa\_ali.fasta


## Gblocks 0.91b Results

Processed file: **SJ15142.aa.fasta.trans.aa\_ali.fasta**  
Number of sequences: **2**  
Alignment assumed to be: **Protein**  
New number of positions: **382** (selected positions are underlined in blue)

```
                         10        20        30        40        50        60
                 =========+=========+=========+=========+=========+=========+
SJ15142          MILRYGGAGAGVGAGSAICLVMVAMKAVQSAASAADRAVLGAVTPDTLGMGEEVRIRWSY
Mac.72377.1      ------------------------------------------------------------
                                                                             


                         70        80        90       100       110       120
                 =========+=========+=========+=========+=========+=========+
SJ15142          DDGNGGSLGKFDIGLYDCGDAFCGQDDCGAFVAGICDHPFDGCGDGQGDYWMTVPEDLTA
Mac.72377.1      ------------------------------------------------------------
                                                                             


                        130       140       150       160       170       180
                 =========+=========+=========+=========+=========+=========+
SJ15142          GDSYKIRVGLHGTELFDCTSTFEIVDRIPVPSMEVFSPRVVLGSESVEDTYEEDDDDASS
Mac.72377.1      ------------------------------------------------------------
                                                                             


                        190       200       210       220       230       240
                 =========+=========+=========+=========+=========+=========+
SJ15142          VVESFSQRRFRHLSTSSSSSEPQLDGEMFGYASTNDTLLVSWAYDDGFQGSFARFNVDLY
Mac.72377.1      ------------------------------------------------------------
                                                                             


                        250       260       270       280       290       300
                 =========+=========+=========+=========+=========+=========+
SJ15142          YCGGACSRAGNCGEMIARLCEACPDSSGSELVTVPDVYSSHNYKIRVSTWSGVRRPASSC
Mac.72377.1      ------------------------------------------------------------
                                                                             


                        310       320       330       340       350       360
                 =========+=========+=========+=========+=========+=========+
SJ15142          SPKFEIRARDRPYYSTPSPTGVPEAEGPSLYPRPGSMAPSEDLPIEPVIRPSVFPDWGEK
Mac.72377.1      ------------------------------------------------------------
                                                                             


                        370       380       390       400       410       420
                 =========+=========+=========+=========+=========+=========+
SJ15142          YVPPAQVACNDSDVPDVRYAGTTKRIYIERNGTRGGCFTLTQIWESGKRPLYPVDPFNNS
Mac.72377.1      ------------------------------------------------------------
                                                                             


                        430       440       450       460       470       480
                 =========+=========+=========+=========+=========+=========+
SJ15142          RVTTPTGVWLLTEELYILDGVTLQLHGTARGGDCDELRLLSDESTILNLRAHGGSLSLLG
Mac.72377.1      --------------------------GTARGGDCDELRLLSDENTILNLRAHGGSLSLLG
                                           ##################################


                        490       500       510       520       530       540
                 =========+=========+=========+=========+=========+=========+
SJ15142          TKVVSWDEGKGTVDTNHEDGRSYISAVSELIYDPSQECNGTAKENRGETRMDVIDSEVGY
Mac.72377.1      TKVVSWDEAKGAVDTNHEDGRSYISAVSELIYDPSQNCNGTAKENRGETRMDVIDSEVGY
                 ############################################################


                        550       560       570       580       590       600
                 =========+=========+=========+=========+=========+=========+
SJ15142          LGYHASESYGLVWKVRGFCIDKSNEEVFAEVNVYGDMINSNIHHNYFGHYSYGHQGGNWS
Mac.72377.1      LGYHESESYGLVWKVRGFCIDKSNEEVFAEVNVYGDMINSNIHHNYFGHYSYGHQGGNWS
                 ############################################################


                        610       620       630       640       650       660
                 =========+=========+=========+=========+=========+=========+
SJ15142          YNKMHDNVEYGFDPHDDSDYLTIVGNDVWGNGNHGIIASKRCDHVTISDNHVSFGVNTGL
Mac.72377.1      YNKMHDNVEYGFDPHDDSDYLTIVGNDVWGNGNHGIIASKRCDHVTISNNHVSFGVNTGL
                 ############################################################


                        670       680       690       700       710       720
                 =========+=========+=========+=========+=========+=========+
SJ15142          FLHRSSDFAIVTGNHVHNNGDAGLAVLESFYMDVHDNVFKDNRYGIRFSVGAGYNWINNN
Mac.72377.1      FLHRSSDFAIVTGNHVHNNGDAGLAVLESFYMDVHDNVFKDNRYGIRFSVGAGYNWINNN
                 ############################################################


                        730       740       750       760       770       780
                 =========+=========+=========+=========+=========+=========+
SJ15142          VINRSFRYNIFTYLGSDLPDVPDTTGFPVGNVFWRNYISGTYLDTIKLKEADLTVIANNR
Mac.72377.1      VINRSFRYNIFTYLGSDLPDVPDTTGFPIGNVFWRNYISGTYLDTIKLKEAGLTLIANNR
                 ############################################################


                        790       800       810       820
                 =========+=========+=========+=========+========
SJ15142          ISSATTIMFDQCTDTTYRDNTQEDYLSFSVRTDSCFSGDSDSDLSPTC
Mac.72377.1      ITSAATIMFDQCTDTTYRDNTQEEYLTFSVRTDSCFSSDSDSDLSPVC
                 ################################################
```

```
Parameters used
Minimum Number Of Sequences For A Conserved Position: 2
Minimum Number Of Sequences For A Flanking Position: 2
Maximum Number Of Contiguous Nonconserved Positions: 8
Minimum Length Of A Block: 10
Allowed Gap Positions: None
Use Similarity Matrices: Yes
```

```
Flank positions of the 1 selected block(s)
Flanks: [447  828]  

New number of positions in SJ15142.aa.fasta.trans.aa_ali.fasta-gb:  382  (46% of the original 828 positions)
```
